# Withdrawal: Influence of lumbar support on tractor seat comfort based on body pressure distribution

**DOI:** 10.1371/journal.pone.0285144

**Published:** 2023-04-25

**Authors:** The PLOS ONE Editors

*PLOS ONE* has withdrawn this article [1] and removed the article contents from the journal website because it was published in error. The publisher apologizes for the error.
